# Handlungsempfehlung zur Therapieumstellung von Immunsuppressiva auf Dupilumab bei Patienten mit atopischer Dermatitis

**DOI:** 10.1007/s00105-020-04720-1

**Published:** 2020-11-11

**Authors:** Johannes Wohlrab, Ulrich Mrowietz, Stephan Weidinger, Thomas Werfel, Andreas Wollenberg

**Affiliations:** 1 Universitätsklinik und Poliklinik für Dermatologie und Venerologie, Martin-Luther-Universität Halle-Wittenberg, Ernst-Grube-Str. 40, 06097 Halle (Saale), Deutschland; 2 An-Institut für angewandte Dermatopharmazie, Martin-Luther-Universität Halle-Wittenberg, Halle-Wittenberg, Deutschland; 3 Klinik für Dermatologie, Venerologie und Allergologie, Christian-Albrechts-Universität Kiel, Kiel, Deutschland; 4 Klinik für Dermatologie und Allergologie, Medizinische Hochschule Hannover, Hannover, Deutschland; 5 Klinik für Dermatologie und Allergologie, Ludwig-Maximilian-Universität München, München, Deutschland

**Keywords:** Antientzündliche Systemtherapie, Langzeittherapie, Pharmakologie, Handlungsempfehlungen, Molekulare Pathogenese, Anti-inflammatory systemic treatment, Long-term treatment, Pharmacology, Treatment recommendations, Molecular pathogenesis

## Abstract

Basierend auf neuen Erkenntnissen zur molekularen Pathogenese der atopischen Dermatitis, wurde neben Glukokortikoiden und Ciclosporin mit Dupilumab nun auch eine zielgerichtete antientzündliche Systemtherapie zugelassen. Wegen ihrer Pharmakologie sind weder Glukokortikoide und Ciclosporin noch die außerhalb der Zulassung angewendeten Substanzen Methotrexat, Azathioprin und Mycophenolsäurederivate für eine Langzeittherapie geeignet. Bei der Umstellung der Therapie von den genannten niedermolekularen Substanzen auf Dupilumab sollten verschiedene Faktoren berücksichtigt werden. Hierbei sind sowohl der konkrete Anlass der Umstellung (Unwirksamkeit, unerwünschte Wirkungen oder sich einstellende Kontraindikationen) als auch die pharmakologischen Gegebenheiten zu berücksichtigen. Da es hierzu bisher keine konkreten klinischen Untersuchungen gibt, haben die Autoren auf der Grundlage einer Literaturrecherche Handlungsempfehlungen für den praktischen Alltag erarbeitet.

## Abstract (en)

Based on new insights into the molecular pathogenesis of atopic dermatitis, a targeted anti-inflammatory therapy—dupilumab—has recently been approved as treatment alongside glucocorticoids and ciclosporin. Due to their pharmacology, neither glucocorticoids nor ciclosporin nor the off label used substances methotrexate, azathioprine and mycophenolic acid derivatives are suitable for long-term therapy. When switching therapy from small molecular substances to dupilumab, various factors should be considered. Both the specific cause of the change (ineffectiveness, adverse effects or contraindications) as well as the pharmacological conditions should be taken into account. Since there have been no specific clinical studies on this subject so far, the authors relied mainly on a literature search to draw up recommendations for practical everyday use.

Die atopische Dermatitis (AD) ist eine chronisch entzündliche Erkrankung, die sich auf der Grundlage einer komplexen genetischen Disposition entwickelt. Sie stellt eine immun-vegetative Dysfunktion dar und ist durch eine Th2-mediierte Entzündung, eine epidermale Barrierefunktionsstörung sowie eine kutane Dysbiose charakterisiert. Für das therapeutische Management der entzündlichen Symptome werden in Abhängigkeit von der Schwere der Erkrankung eine konsequente barriereprotektive Basistherapie [1], eine UV(Ultraviolett)-Therapie [2–4] bzw. eine topische und/oder systemische Pharmakotherapie eingesetzt [5]. Für die topische Applikation stehen dabei Glukokortikoide (TGK) [6] in unterschiedlichen Wirkstärken, mit einem variablen therapeutischen Index [7, 8] und in unterschiedlichen Galeniken sowie verschiedene Calcineurininhibitoren (TCI) [9] zur reaktiven und proaktiven Therapie zur Verfügung. In der Systemtherapie können in Ausnahmefällen Glukokortikoide (SGK), indikationsgerecht Ciclosporin (CSA) [10] oder Dupilumab [11], und als Off-label-Therapien Methotrexat (MTX) [12, 13], Azathioprin (AZA) [14–17] oder Mycophenolsäurederivate (MPA) [18] eingesetzt werden. Für ein chronisches Handekzem im Rahmen des atopischen Syndroms kann auch eine Therapie mit Alitretinoin (ALI) erwogen werden [19].

Es gibt verschiedene Szenarien, die dazu führen können, dass eine immunsuppressive Systemtherapie mit den genannten kleinmolekularen Arzneistoffen beendet und auf eine zielgerichtete Therapie mit dem therapeutischen Antikörper Dupilumab umgestellt werden muss. Für das dabei aus pharmakologischer und regulatorischer Sicht notwendige Management gibt es aber aktuell nur sehr pauschale und wenig differenzierte Handlungsempfehlungen bzw. systematische Entscheidungshilfen [20]. Diese wurden nun durch eine Expertengruppe bearbeitet und sollen im Folgenden verfügbar gemacht werden.

## Pharmakologische Kenndaten relevanter Arzneistoffe

### Arzneistoffe mit Zulassung in der Indikation „atopische Dermatitis“

Für die Systemtherapie der atopischen Dermatitis sind in Deutschland nur SGK, CSA und Dupilumab zugelassen [21]. Beim Einsatz von SGK und CSA sollte wegen der immunsuppressiven Wirkung ein erhöhtes Risiko für Infektionen beachtet werden [22]. Ergänzend dazu gilt eine Kontraindikation für Lebendimpfstoffe. Zudem sollte der Einsatz bei manifesten oder anamnestischen Malignomen vermieden werden. Es liegt für keinen der genannten Wirkstoffe eine Zulassung für den Einsatz in der Schwangerschaft oder Stillzeit vor. Aufgrund umfangreicher Daten kann aber der Einsatz, insbesondere von CSA, nach einer individuellen Risikoabschätzung und mit Einverständnis der Patientin erwogen werden [23].

#### Glukokortikoide

Bei SGK handelt es sich um lipophile Hormone, die eine genomische (zytosolische Rezeptorbindung nach transmembranöser Diffusion) und eine nichtgenomische Wirkung (Interaktionen mit Membranen) entfalten. Die empfohlene Tagesdosis von SGK bei atopischer Dermatitis ist nicht einheitlich und richtet sich nach der Schwere der Erkrankung und möglicher Komorbidität bzw. Komedikation. Im klinischen Alltag hat sich eine Dosierung von 0,5–1,0 mg/kgKG [Körpergewicht]/Tag Prednisolonäquivalent bis maximal 14 Tage bewährt, um ein Ausschleichmanöver zur Dosisreduktion zu vermeiden. Eine Anwendung für den mittel- und langfristigen Gebrauch bei atopischer Dermatitis kann wegen der unerwünschten Arzneimittelwirkungen (UAWs) nicht empfohlen werden [5]. So geht der Einsatz von SGK selbst bei niedrig dosierter Anwendung regelmäßig mit unerwünschten Effekten einher. Das Ausmaß und die Reversibilität sind interindividuell sehr verschieden und stark abhängig von Dosierung, Anwendungsdauer und Applikationsform. Unter Langzeitanwendung zeigen sich Suppressionsphänomene der adrenalen Achse, Störungen des Kalziumstoffwechsels mit Osteoporose, zentralnervöse Symptome, Myopathie, Blutbildveränderungen, Wachstumsretardierung im Kindesalter, Kataraktentwicklung, Steroiddiabetes sowie gastrointestinale Ulkusbildung [24, 25]. Sollte eine mittel- und langfristige Systemtherapie notwendig sein, so empfiehlt sich leitliniengerecht entweder die Einleitung einer CSA- oder einer Dupilumab-Therapie [26, 27].

#### Ciclosporin

Ciclosporin (CSA) ist ein stark lipophiles zyklisches Polypeptid. Es bindet nach Diffusion durch die Zellmembran (z. B. Lymphozyten) an das zytoplasmatische Rezeptormolekül Cyclophilin (Familie der Immunophiline), inhibiert dadurch die kalziumabhängige Phosphatase Calcineurin und damit die Aktivierung eines nukleären Faktors aktivierter T‑Zellen (NFAT), der nach Translokation in den Zellkern die Genaktivierung und Transkription von proinflammatorischen Zytokinen (z. B. IL[Interleukin]-2, TNF[Tumor-Nekrose-Faktor]-α, IFN[Interferon]-γ) reguliert [28]. Es wird typischerweise in einem Dosisbereich von 2,5–5 (7,5) mg/kgKG/Tag in 2 Einzeldosen per os verabreicht. Durch den früheren intensiven Einsatz von CSA in der Transplantationsmedizin liegen umfangreiche pharmakologische Daten und Erfahrungen vor. Dies betrifft auch den Einsatz in der Langzeitanwendung, im Kindesalter und in der Schwangerschaft. Wegen der Metabolisierung von CSA in Leber und Darm durch CYP3A4 sowie der Abhängigkeit der Clearance von P‑Glykoprotein treten Arzneimittelinteraktionen mit einer Vielzahl anderer Wirksubstanzen (z. B. Azolantimykotika, Antiepileptika, Terbinafin) auf [29].

Neben einer stark eingeschränkten Nierenfunktion, dem Vorliegen von Malignomen (auch anamnestisch) und einem ausgeprägten arteriellen Hypertonus gelten v. a. schwere Infektionserkrankungen als absolute Kontraindikation. Die kombinierte Exposition mit UVA-Licht (natürliche oder künstliche Bestrahlung) oder die einer Ciclosporin vorausgehenden PUVA-Therapie gelten als Risikofaktor für die Entstehung von nichtmelanozytärem Hautkrebs [30–32].

Unter der Therapie werden gelegentlich nach einer Therapiedauer von >2 Jahren häufig Nierenfunktionsstörungen und die Ausbildung eines arteriellen Hypertonus beobachtet [31, 33–35]. Seltener treten eine Gingivahyperplasie oder eine Hypertrichose auf [36]. Die Verträglichkeit im Kindesalter scheint besser zu sein als bei Erwachsenen [37–39]. Das in der Transplantationsmedizin indizierte „drug monitoring“ ist in der Dermatologie pharmakologisch weder notwendig noch sinnvoll und wird nur zur Überprüfung der Therapieadhärenz genutzt [40].

#### Dupilumab

Mit Dupilumab steht für erwachsene Patienten seit 2017 und für Jugendliche (ab 12. Lebensjahr) seit 2019 ein therapeutischer Antikörper für die zielgerichtete Therapie der mittelschweren bis schweren AD zur Verfügung. Er bindet an die α‑Untereinheit des IL-4-Rezeptors und inhibiert damit die Aktivierung der intrazellulären Signalkaskade von IL‑4 und IL-13 [41]. Dupilumab ist ein Ig(Immunglobulin)G-Molekül, das ca. 7 bis 10 Tage nach subkutaner Applikation die höchste Serumkonzentration erreicht und eine absolute Bioverfügbarkeit von ca. 64 % aufweist. Es unterliegt wie alle IgG-Moleküle einer Clearance, die durch lineare und nichtlineare Eliminationsprozesse bedingt wird. Neben der zielantigenvermittelten Elimination (sog. „antigen sink“) und einer geringen Proteolyserate in der Leber erfolgt die Elimination v. a. über das retikuloendotheliale System in der Interaktion mit Makrophagen und Monozyten sowie durch unspezifische Pino‑/Endozytose von Endothelzellen, die auch in Abhängigkeit von der Expression des nFcR (Brambell-Rezeptor) einen Recycling-Prozess steuern [42]. Eine Bildung von endogenen Antikörpern gegen Dupilumab (sog. „anti drug antibodies“ [ADA]) mit neutralisierender Wirkung findet sich nur in einem sehr geringen und klinisch nicht relevanten Ausmaß von ca. 0,5–2 % [43]. Da bei der Anwendung von Dupilumab keine direkten Arzneimittelinteraktionen mit einem der bereits genannten „small molecules“ zu erwarten sind, bestehen aus pharmakologischer Sicht keine Bedenken gegen eine unmittelbare, indikationsgerechte Anschlusstherapie bzw. eine kombinierte Anwendung von Dupilumab mit einem konventionellen Immunsuppressivum wie SGK, CSA, MTX, AZA oder MPA. Die vorliegenden klinischen Studiendaten mit Dupilumab (SOLO1 und 2, CHRONOS) weisen bei der Mehrzahl der Patienten einen soliden therapeutischen Effekt (klinische relevante Reduktion von EASI [Eczema Area and Severity Index]/SCORAD [Scoring Atopic Dermatitis] bereits nach ca. 4 bis 6 Wochen) aus. Eine klinisch relevante Reduktion des Juckreizes wird häufig schon früher beobachtet [44–46]. Auch wenn die Ansprechraten im weiteren Therapieverlauf noch leicht ansteigen, so kann bei der Mehrzahl der Patienten davon ausgegangen werden, das nach dieser Initialphase der Dupilumab-Anwendung ein relevantes klinisches Ansprechen eintritt [43, 47]. Dupilumab wird häufig bei Patienten initiiert, die sich noch unter einer Therapie mit Immunsuppressiva, insbesondere CSA, befinden. Für eine solche Therapieumstellung von CSA auf Dupilumab kann eine kombinierte Anwendung für 4 bis 6 Wochen erwogen werden. Für eine längerfristige kombinierte Anwendung eines konventionellen Immunsuppressivums mit Dupilumab gibt es bisher keine Evidenz.

### Arzneistoffe, die in der Indikation atopische Dermatitis „off label“ angewendet werden

#### Methotrexat

Das Zytostatikum MTX wird nach aktiver Aufnahme in die Zelle glutamyliert und hemmt die Dihydrofolatreduktase (Antifolat), die als Schlüsselenzym der Folsäuresynthese fungiert und somit eine Voraussetzung für die RNA(Ribonukleinsäure)- und DNA(Desoxyribonukleinsäure)-Synthese schafft. Dadurch werden antiproliferative und antiinflammatorische Effekte vermittelt [48]. Für die Anwendung bei Patienten mit atopischer Dermatitis im Dosisbereich zwischen 10 und 15 (25) mg/Woche p.o./s.c. gibt es mittlerweile eine gute klinische Evidenz [12]. Dies trifft insbesondere für die Anwendung im Kindesalter zu [13, 49–51]. Zudem liegen direkte Studienvergleichsdaten zu CSA und AZA vor [52–54]. Unter der Anwendung von MTX werden verschiedene Arzneimittelinteraktionen und UAWs beobachtet [55, 56]. Von besonderer klinischer Bedeutung sind dabei gastrointestinale Symptome mit Übelkeit und Schwindel, die häufig auch zum Abbruch der Therapie Anlass geben. Die subkutane Applikation sollte aus pharmakokinetischen und Sicherheitsgründen der oralen Gabe vorgezogen werden [57]. Durch die Gabe von 5 mg Folsäure 24 h nach MTX-Applikation kann Blutbildungsstörungen (insbesondere Agranulozytose) vorgebeugt werden [58, 59]. Besondere Beachtung sollte bei jungen männlichen Patienten die Beratung hinsichtlich der Reduktion der Fertilität erfahren [60, 61].

#### Azathioprin

Azathioprinwird durch das Enzym Glutathion-S-Transferase metabolisiert. Der dabei entstehende Metabolit 6‑Mercaptopurin passiert die Zellmembran und wird wesentlich durch die Thiopurinmethyltransferase (TPMT) weiter in verschiedene Metabolite gespalten, die die DNA- und RNA-Synthese und somit die Klonalisierung von T‑ und B‑Zellen hemmen [62]. Die daraus resultierende immunsuppressive Wirkung wird in Abhängigkeit von der individuell variablen Expression des Genotyps der TPMT und deren daraus resultierender Aktivität im Dosisbereich von 1–3 mg/kgKG/Tag AZA (in 1 bis 3 Einzeldosen) erzielt [17, 63]. Als relevante UAWs gelten Leukozytopenie, Thrombopenie und Anämie (Myelotoxizität), Infektanfälligkeit, Überempfindlichkeitsreaktionen bzw. Leber- und Nierenfunktionsstörungen sowie in Assoziation mit UV-Licht ein erhöhtes Risiko für nichtmelanozytären Hautkrebs. Männliche Patienten sollten während und bis 6 Monate nach Therapie keine Kinder zeugen [62].

#### Mycophenolsäurederivate

Mycophenolat-Mofetil (MMF) und Mycophenolnatrium (MPN) sind 2 Prodrugs der MPA, die ihre Wirkung durch Hemmung der Inosinmonophosphatdehydrogenase, einem bedeutsamen Enzym bei der DNA-Synthese, insbesondere in Lymphozyten entfaltet [64, 65]. MMF wird bei der AD im Dosisbereich von 2‑mal/Tag 0,5–1,0 g p.o. und MPN 2‑mal/Tag 720 mg p.o. eingesetzt [66]. Beide Prodrugs gelten hinsichtlich ihrer Wirksamkeit in den genannten Dosisbereichen bei der AD als weitgehend äquivalent. Bezüglich potenzieller UAWs sollte v. a. die teratogene Wirkung der MPA beachtet werden [67]. Darüber hinaus können unter der Anwendung Infektionen, Blutbildungsstörungen (insbesondere Anämie und Thrombopenie) sowie gastrointestinale Symptome auftreten.

#### Alitretinoin

Der Einsatz von ALI wird insbesondere im Rahmen eines chronischen Handekzems praktiziert [68, 69]. Allerdings liegen auch Daten zum therapeutischen Nutzen bei AD vor [19]. Die Wirksamkeit von ALI bei AD lässt sich durch die duale agonistische Wirkung auf den Retinsäurerezeptor (RAR) und den Retinoid-X-Rezeptor (RXR) ableiten [70]. ALI wirkt wie alle Retinoide teratogen und darf nicht bei Schwangeren eingesetzt werden [71].

## Szenarien für einen Wechsel der Systemtherapie

Für einen Therapiewechsel können mehrere Ursachenkonstellationen vorliegen, die ein objektives oder subjektives Hindernis zur Fortsetzung einer etablierten Therapie darstellen.

Eine ungenügende Wirksamkeit liegt dann vor, wenn nach ausreichend langer Anwendungsdauer (ca. 12 bis 16 Wochen) in einer ausreichend hohen Dosis (empfohlener Dosisbereich entsprechend Zulassung) keine oder eine zu geringe Wirksamkeit (z. B. Nichterreichen eines EASI-50) festgestellt werden muss. Zudem kann auch nach längerfristiger, wirksamer Therapie eine sekundäre Wirkminderung eintreten, die zum Therapiewechsel veranlasst. Darüber hinaus können unter der Pharmakotherapie spezifische oder unspezifische unerwünschte Wirkungen (z. B. Infektion) eintreten bzw. sich Kontraindikation für die Fortsetzung der Therapie (z. B. Kinderwunsch, Schwangerschaft) ergeben [71]. UAWs können schwerwiegend sein und obligat einen Therapieabbruch notwendig machen. Meistens stellen sich aber befristet tolerierbare bzw. kontrollierbare UAWs ein, die dennoch einer mittel- und langfristigen Therapiefortsetzung entgegenstehen und einen Therapiewechsel begründen.

Bei der Auswahl einer klinisch und pharmakologisch fundierten Handlungsstrategie müssen mehrere Faktoren beachtet werden. Kern der Überlegungen stellt hierbei das Bemühen dar, Schaden vom Patienten abzuwenden. Die Risiken liegen dabei sowohl im Eintreten unerwünschter Arzneimittelwirkungen als auch in der Zunahme der Krankheitsaktivität (schnelles Rezidiv) während des Therapiewechsels. Im Folgenden werden typische klinische Situationen dargestellt und Handlungsempfehlungen formuliert. Die Empfehlungen basieren mangels klinischer Daten auf einer nichtsystematischen Literaturrecherche, auf pharmakologischen Erwägungen sowie auf den Erfahrungen der Experten.

### Grundsätzliches

Hinsichtlich eines Therapiewechsels von kleinmolekularen Arzneistoffen (CSA, MTX, AZA oder MMF/MPN) auf einen therapeutischen Antikörper (mAb) muss zunächst grundsätzlich festgestellt werden, dass dessen Pharmakokinetik ein völlig unterschiedliches Eliminationsverhalten zugrunde liegt [42]. Während kleinmolekulare Arzneistoffe durch renale oder hepatische Elimination bzw. durch Metabolisierung in anderen Geweben inaktiviert werden, erfolgt die Degradierung von mAb v. a. durch Bindung an das Zielepitop („antigen sink“), durch Proteolyse nach Aufnahme durch Zellen des retikuloendothelialen Systems (z. B. Makrophagen) bzw. durch unspezifische Endozytose (z. B. durch Endothelzellen) und anschließende lysosomale Zersetzung. Eine chemische Interaktion zwischen kleinmolekularen Arzneistoffen und mAb ist somit extrem unwahrscheinlich und besitzt keine praktische Relevanz. Eine Koapplikation der beiden Substanzgruppen ist somit aus pharmakokinetischer Sicht unkritisch [72].

### Therapie mit Ciclosporin

Ciclosporin (CSA) ist eine seit Langem bekannte und pharmakologisch gut charakterisierte Substanz. Multiple Studien aus der Transplantationsmedizin sowie den Phase-III- und -IV-Studien in der Indikation AD zeigen, dass CSA bei AD eine rasch wirksame und bei internistisch gesunden Patienten auch gut verträgliche Substanz darstellt. Dennoch ist bekannt, dass insbesondere bei multimorbiden bzw. älteren Patienten nach ca. 1 bis 2 Jahren Anwendungsdauer (insbesondere bei Einsatz hoher Dosen von 5,0 mg/kgKG/Tag) das Risiko der Entwicklung von UAWs relevant zunimmt. Diese Beobachtung hat zum einen dazu geführt, dass nach dem Ansprechen und Stabilisieren der Krankheitsaktivität eine Dosisreduktion auf 2,5 mg/kgKG/Tag angestrebt werden sollte, zum anderen die Empfehlung ausgesprochen wird, nach 2‑jähriger Anwendung auch bei Ausbleiben von UAWs die Therapie nach Möglichkeit auf eine andere Erhaltungstherapie umzustellen [26]. Eine besondere Situation ergibt sich bei Kindern, die deutlich seltener als Erwachsene relevante UAWs entwickeln und bei denen bis zum Alter von 12 Jahren aktuell auch keine zugelassenen Therapiealternativen existieren [38].

Ciclosporin muss insbesondere bei laborchemischen Hinweisen auf eine nephrotoxische UAW in der Dosis reduziert oder abgesetzt werden. Steigt der Kreatiningehalt im Serum um mehr als 30 % vom Ausgangswert (auch wenn dieser im Normbereich liegt) und persistiert über 2 Wochen, sollte die Dosis um 1 mg/kgKG/Tag reduziert werden. Kommt es darunter auch nach 4 Wochen zu keinem relevanten Kreatininabfall, sollte die Dosis weiter um 1 mg/kgKG/Tag reduziert bzw. bei weiterer Persistenz des Kreatininanstieges abgesetzt werden. Tritt hingegen eine Kreatininsteigerung von >50 % des Ausgangswertes auf, sollte die Dosis halbiert und nach 2 bis 4 Wochen ohne Kreatininabfall abgesetzt werden. Bei bekannter Vorschädigung der Nierenfunktion sollte eine maximale Initialdosis von 2,5 mg/kgKG/Tag zur Anwendung kommen. Auch die Entwicklung einer arteriellen Hypertonie stellt eine bekannte UAW dar. Diese kann ggf. durch eine antihypertensive Therapie korrigiert werden. Stellen sich hier therapierefraktäre hypertensive Werte ein, muss die Therapie abgebrochen werden.

**Figure 1:**
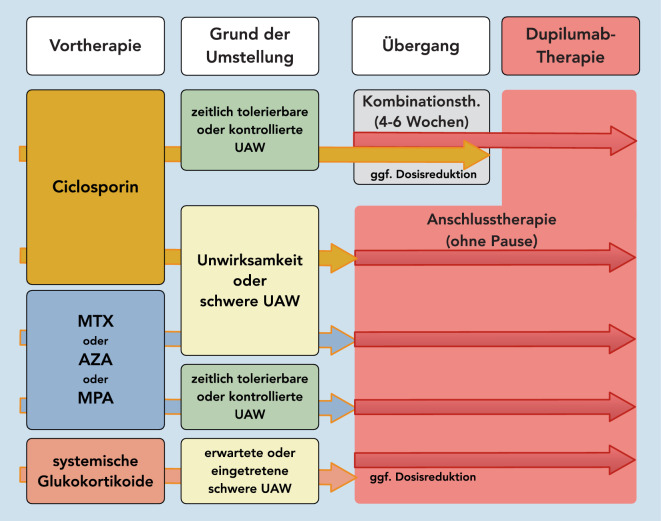

Für die Umstellung der Therapie auf einen mAb (z. B. Dupilumab) ist zu beachten, dass bei Unwirksamkeit von CSA (Nichterreichen eines EASI-50) in einer Dosierung von 5 mg/kgKG/Tag über 12 bis 16 Wochen bzw. bei Eintreten von nicht zeitlich tolerierbaren oder kompensierten UAWs CSA abgesetzt werden und sofort anschließend, ohne Therapiepause, die Einstellung auf Dupilumab erfolgen sollte (Abb. 1). Erfolgt die Therapieumstellung auf Dupilumab aber wegen zeitlich tolerierbarer oder gar kompensierter UAWs bzw. bei einer CSA-Anwendung über 1 bis 2 Jahre zur Vermeidung von UAWs, sollte CSA in der etablierten Dosis für 4 bis 6 Wochen überlappend zur Dupilumab-Therapie gegeben werden, um ein schnelles Rezidiv der AD zu vermeiden (Abb. 1).

### Therapie mit Methotrexat, Azathioprin oder Mycophenolsäurederivaten

Bei den nicht zur Behandlung der AD zugelassenen Arzneistoffen MTX, AZA und MMF/MPN sollte beachtet werden, dass bei Unwirksamkeit bzw. beim Auftreten relevanter UAWs ein Absetzen ohne Ausschleichen erfolgt. Auch hier ist unmittelbar im Anschluss (ohne Therapiepause) der Beginn einer Dupilumab-Applikation möglich. Eine zeitliche Überlappung durch Kombinationsgabe über 4 bis 6 Wochen ist zwar pharmakokinetisch unkritisch, erscheint aber wegen der längeren Effekthalbwertszeiten nicht sinnvoll, da ein schnelles Rezidiv nicht zu erwarten ist. Anders ist der Umstand zu bewerten, wenn im Vorfeld noch keine CSA-Gabe erfolgte und ein Umstellen auf CSA angestrebt wird.
